# Voltammetric Evidence of Proton Transport through the Sidewalls of Single-Walled Carbon Nanotubes

**DOI:** 10.1021/jacs.3c00554

**Published:** 2023-03-28

**Authors:** Jack W. Jordan, Beth Mortiboy, Andrei N. Khlobystov, Lee R. Johnson, Graham N. Newton, Darren A. Walsh

**Affiliations:** † Nottingham Applied Materials and Interfaces (NAMI) Group, GSK Carbon Neutral Laboratories for Sustainable Chemistry, School of Chemistry, University of Nottingham, Nottingham NG7 2TU, U. K.; ‡ The Faraday Institution, Quad One, Harwell Science and Innovation Campus, Didcot OX11 0RA, U. K.; § School of Chemistry, University of Nottingham, Nottingham NG7 2RD, U. K.

## Abstract

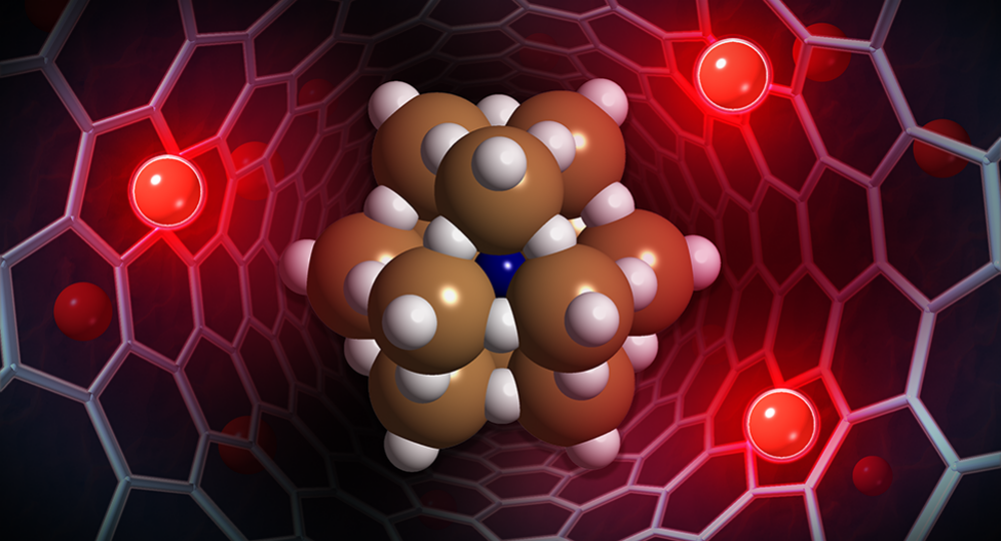

Understanding ion transport in solid materials is crucial in the design of electrochemical devices. Of particular interest in recent years is the study of ion transport across 2-dimensional, atomically thin crystals. In this contribution, we describe the use of a host–guest hybrid redox material based on polyoxometalates (POMs) encapsulated within the internal cavities of single-walled carbon nanotubes (SWNTs) as a model system for exploring ion transport across atomically thin structures. The nanotube sidewall creates a barrier between the redox-active molecules and bulk electrolytes, which can be probed by addressing the redox states of the POMs electrochemically. The electrochemical properties of the {POM}@SWNT system are strongly linked to the nature of the cation in the supporting electrolyte. While acidic electrolytes facilitate rapid, exhaustive, reversible electron transfer and stability during redox cycling, alkaline-salt electrolytes significantly limit redox switching of the encapsulated species. By “plugging” the {POM}@SWNT material with C_60_-fullerenes, we demonstrate that the primary mode of charge balancing is proton transport through the graphenic lattice of the SWNT sidewalls. Kinetic analysis reveals little kinetic isotope effect on the standard heterogeneous electron transfer rate constant, suggesting that ion transport through the sidewalls is not rate-limiting in our system. The unique capacity of protons and deuterons to travel through graphenic layers unlocks the redox chemistry of nanoconfined redox materials, with significant implications for the use of carbon-coated materials in applications ranging from electrocatalysis to energy storage and beyond.

## Introduction

Two- and three-dimensional nanocarbons have been used as electrically conductive supports and electrode materials for electrochemical devices, leading to increasing interest in the study of ion-transport in such materials.^1−7^ Recently, Geim and co-workers demonstrated that field-driven migration of protons through atomically thin, pristine monolayers of graphene, and hexagonal boron nitride (h-BN) was possible.^8^ Ion conductivities between 1 and 10^4^ mS cm^–2^ have since been reported,^1,9^ while through-layer transport of larger ions, such as Li^+^, has not been detected.^10^ The rate of proton transport through graphene exceeds that of deuterons by an order of magnitude,^11,12^ and monolayer graphene may be used as a selective proton-conducting membrane in next-generation devices.^13^

The ion-transport properties of 2D materials have been studied using specialized experimental setups, such as Nafion|graphene|Nafion sandwich structures,^8^ membrane-electrode assemblies,^10^ and “proton-pump” systems, in which the graphene sheet is modified with hydrogen-evolution catalysts on one face.^14^ In contrast, the ion-transport and redox properties of 3D, solid-state materials have been studied routinely for decades using electrochemical methods such as voltammetry and galvanostatic cycling.^15−17^ These studies have been extended to 2D and 3D nanocarbons, including graphene and single-walled carbon nanotubes (SWNTs), which can be thought of as rolled-up sheets of graphene. The fundamental properties of these materials, as well as their applications in devices,^18−25^ have been studied electrochemically, revealing insights into the physicochemical properties of these systems. These include the effects of dopants and heteroatoms on the kinetics of electron transfer at graphene-based electrodes.^26^ A remarkable feature of SWNTs is that they can be modified with redox- and catalytically active materials in their interiors; a lot of recent research has focused on the chemical and electrochemical properties of chemical species “nanoencapsulated” within SWNTs, which often imparts unique properties to the resultant materials,^27,28^ such as novel electronic properties,^29−31^ chemical stability,^27^ and tunable catalytic selectivity.^32^

Polyoxometalates (POMs) are polyanionic molecular metal oxide clusters based on early transition metals that have attracted attention in recent years as charge carriers and redox mediators in a range of technologies.^33−37^ In this contribution, we describe the use of POMs nanoencapsulated within SWNTs as a probe for studying the transport of ions through the sp^2^ carbon layer. The Wells-Dawson phosphotungstate [P_2_W_18_O_62_]^6–^ can be encapsulated within SWNTs^22,38^ and undergoes several consecutive, chemically reversible reductions during voltammetry in the solid state. We show that electrochemical switching between the various redox states is only possible in acidic electrolytes; in the presence of electrolytes containing Li^+^ and Na^+^ ions, the waves associated with redox of the POMs are suppressed. We probe the differences in these voltammetric behaviors by plugging the ends of the SWNTs with size-matched fullerenes, limiting the only possible ion-transport route to the sidewalls of the SWNTs, as well as by using deuterated and nondeuterated supporting electrolytes. Using these strategies, we show that charge-balancing ion transport in the system is achieved by through-wall migration of protons and deuterons.

## Results and Discussion

**Figure 1:**
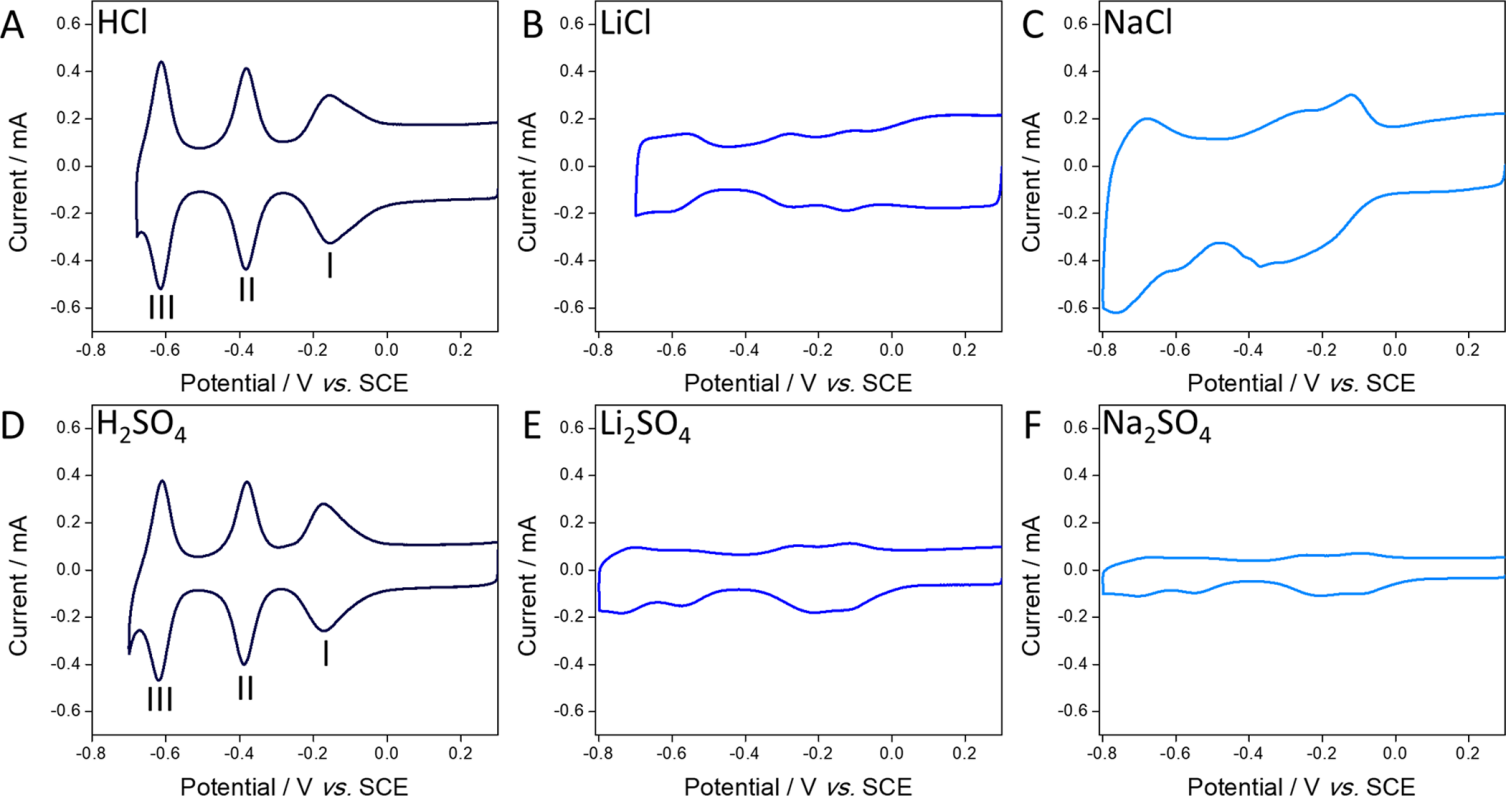
(A–F) Cyclic voltammograms of {P_2_W_18_}@SWNT recorded using 1.0 M aqueous supporting electrolytes (labeled inset). All voltammograms were recorded using a glassy carbon working electrode, SCE reference electrode, glassy carbon counter electrode, and scan rate of 100 mV s^–1^.

[P_2_W_18_O_62_]^6–^ was encapsulated within SWNTs with an average internal diameter of 1.5 nm using the method previously reported.^22,38^ Briefly, the opened SWNTs were added to 10 mM aqueous K_6_[P_2_W_18_O_62_] and stirred for 48 h, after which {P_2_W_18_}@SWNT was isolated by filtration. The POMs were encapsulated without the corresponding counter cations, with the host SWNT effectively balancing the excess charge on the POMs.^22,38,39^ The redox chemistry of {P_2_W_18_}@SWNT was assessed by voltammetric analysis of films of the material immobilized on a glassy carbon (GC) working electrode. Figure 1A shows a cyclic voltammogram (CV) of {P_2_W_18_}@SWNT recorded using 1.0 M HCl as a supporting electrolyte and at 100 mV s^–1^. Three redox couples (I, II, and III) with mid-point potentials, *E*_mid_, of −0.160, −0.382, and −0.612 V were visible in the CV. The peak-to-peak separation (Δ*E*_p_) for couples I, II, and III were 4, 0, and 0 mV, respectively, and the peak currents, *i*_p_, for the reduction process of each couple increased linearly with increasing scan rate, υ (Figure S1, Supporting Information). These observations are expected for electrochemically reversible surface-confined redox processes^15^ and are in good agreement with our previous reports of the electrochemistry of these materials.^22,38^

We investigated the transport of different ions of the supporting electrolyte by first varying the anions of the supporting electrolytes. Figure 1D shows a CV of {P_2_W_18_}@SWNT in contact with 1.0 M H_2_SO_4_, which is similar to the voltammogram recorded in 1.0 M HCl. Three redox couples with mid-point potentials of −0.173, −0.385, and −0.613 V and Δ*E*_p_ values of 1, 7, and 6 mV were observed for couples I, II, and III, respectively. *i*_p_ for each reduction process increased linearly as υ increased (Figure S2, Supporting Information). In contrast, when altering the cation of the supporting electrolyte, by using 1.0 M LiCl, NaCl, Li_2_SO_4_, and Na_2_SO_4_ as supporting electrolytes (Figure 1B,C,E,F, respectively) electrochemical redox of the POMs was suppressed; in each case, lower faradaic currents and ill-defined peaks were observed. Suppression of the electrochemical responses in the Li^+^-containing electrolytes differs from that observed when the POM cluster is dissolved in solution and reversible electroreduction occurs readily.^40,41^

**Figure 2:**
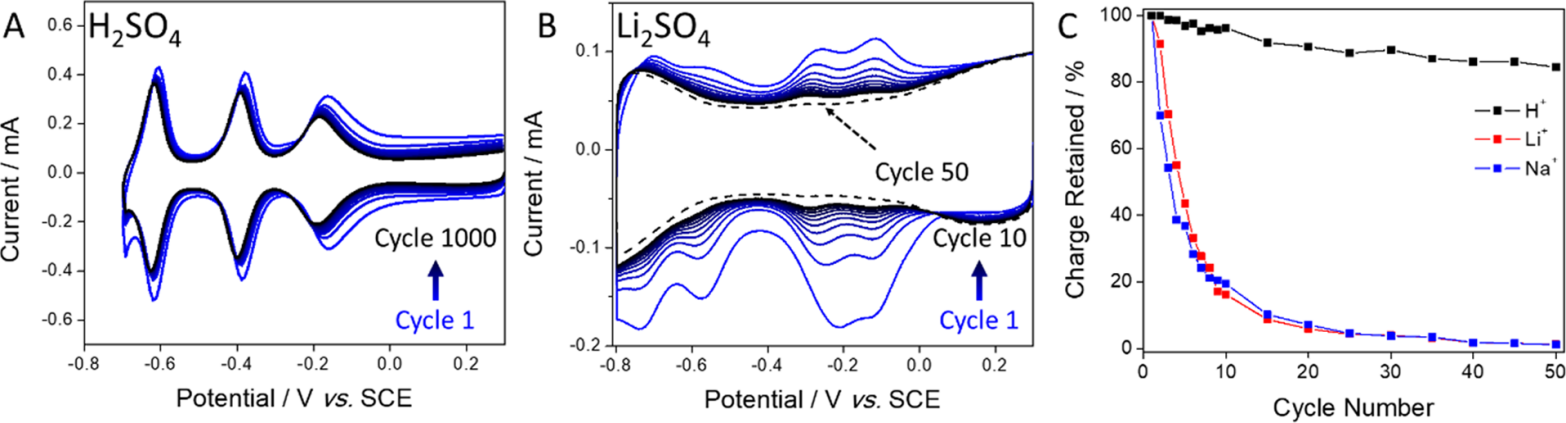
(A) Cyclic voltammograms (cycles 1–1000, every 100th cycle, labeled inset) of {P_2_W_18_}@SWNT recorded using 1.0 M H_2_SO_4_ as supporting electrolyte, showing that the charge passed was retained up to at least the 1000th cycle. (B) Cyclic voltammograms (cycles 1–10 and 50, labeled inset) of {P_2_W_18_}@SWNT recorded using 1.0 M Li_2_SO_4_ as electrolyte and showing a decay in the charge passed over 50 cycles. (C) Charge retention during reduction of couple II during potential cycling of {P_2_W_18_}@SWNT in 1.0 M H_2_SO_4_, Li_2_SO_4_, and Na_2_SO_4_ (labeled inset). All CVs were recorded using a glassy carbon working electrode, SCE reference electrode, glassy carbon counter electrode, and scan rate of 100 mV s^–1^.

The retention in the faradaic charge passed during the first reduction of couple II upon voltammetric cycling in 1.0 M H_2_SO_4_ (Figure 2A) was 85 and 83% after 50 and 1000 cycles, respectively. In contrast, almost no faradaic charge was passed by cycle 10 when cycling the material in 1.0 M Li_2_SO_4_ (Figure 2B) and, by the 50th cycle, the CV showed an almost completely capacitive response. The same rapid decay in the amount of charge passed was also observed when using 1.0 M Na_2_SO_4_ as a supporting electrolyte (Figure 2C), demonstrating that charge-balancing upon redox of the nanoencapsulated POMs depended strongly on the nature of the cations of the supporting electrolyte.

**Scheme 1:**
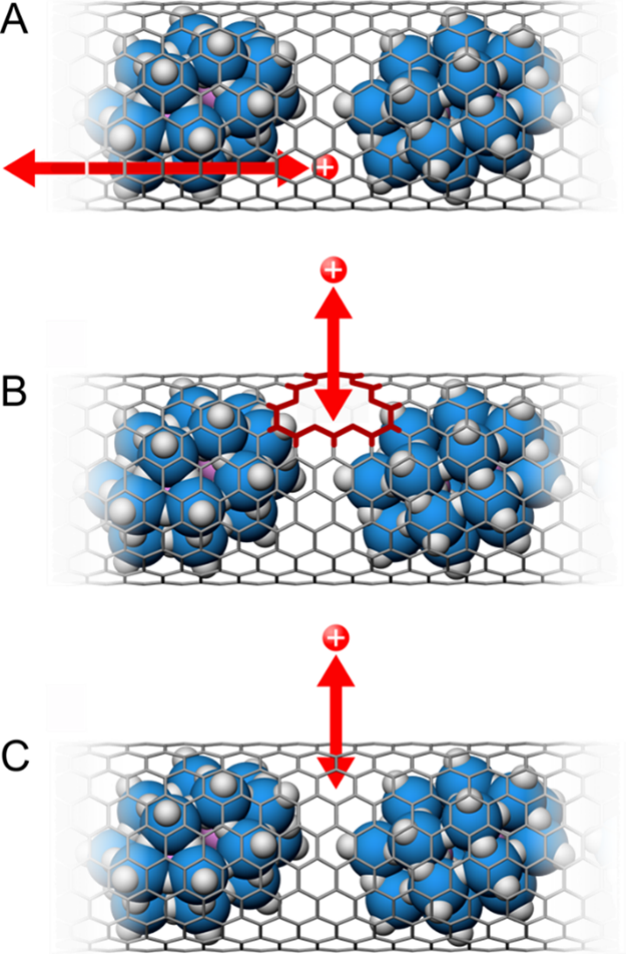
Potential Charge-Balancing Routes upon Redox of Nanoencapsulated [P_2_W_18_O_62_]^6–^ (A) Ion transport along the SWNT internal channel; (B) ion transport through a vacancy defect (highlighted in red); (C) ion transport through the sp^2^ graphenic lattice. POMs are depicted in blue and cations are red point charges for clarity. Ion-transport routes are shown by red arrows.

The potential routes by which the charge-balancing counterions could access the nanoencapsulated redox species during electrochemical cycling are through the open SWNT ends, defects in the SWNT sidewalls, and the sp^2^ carbon framework of the sidewalls (Scheme 1). Based on a recent study estimating the defect density of SWNTs from Raman spectroscopy,^42^ the concentration of defects within our samples is expected to be about 1 per 65 nm (while other reports put the number as low as 1 per 4 μm).^43^ At this density, transport through defects is unlikely to be a significant contributor to charge balancing in the system. In addition, the fact that redox of the POMs was suppressed in the presence of the alkaline metal countercations indicates that defect-facilitated transport did not play a significant role.

**Figure 3:**
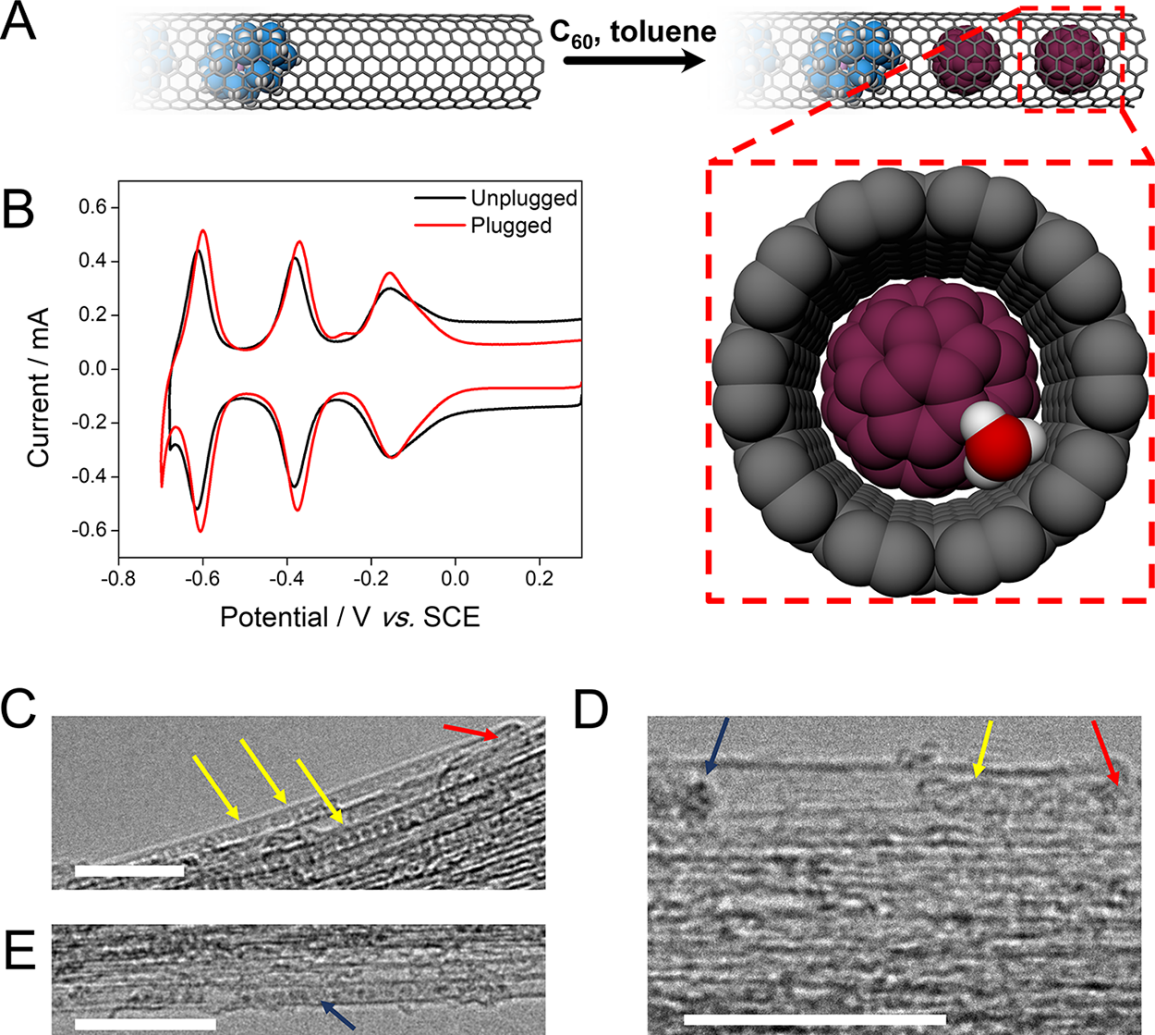
(A) Schematic of the plugging of {P_2_W_18_}@SWNT (POMs shown in blue) with C_60_ fullerenes (purple). The C_60_ molecules are encapsulated within the open SWNT ends, restricting ion access along the main SWNT axis. The expanded view (below, right) shows the end-on view of the plugged SWNT (⊂C_60_{P_2_W_18_}@SWNT) with a hydronium ion (oxygen atom red, hydrogen atoms white) for scale, demonstrating that the van der Waals gap between the C_60_ and SWNT sidewall restricts ion access. (B) Cyclic voltammograms of {P_2_W_18_}@SWNT and ⊂C_60_{P_2_W_18_}@SWNT recorded using 1.0 M HCl as a supporting electrolyte. TEM images of the encapsulation of C_60_ fullerenes using the drop-cast methodology for an SWNT film and a {P_2_W_18_}@SWNT film are shown in C and D, respectively. A TEM image of {P_2_W_18_}@SWNT not subjected to the plugging process is shown in E. The scale bars are 10 nm long and the images were acquired with an accelerating voltage of 100 kV. Yellow arrows show encapsulated fullerenes, red arrows show SWNT open ends, and the blue arrows show encapsulated [P_2_W_18_O_62_]^6–^. All voltammograms were recorded using a glassy carbon working electrode, SCE reference electrode, glassy carbon counter electrode, and a scan rate of 100 mV s^–1^.

**Figure 4:**
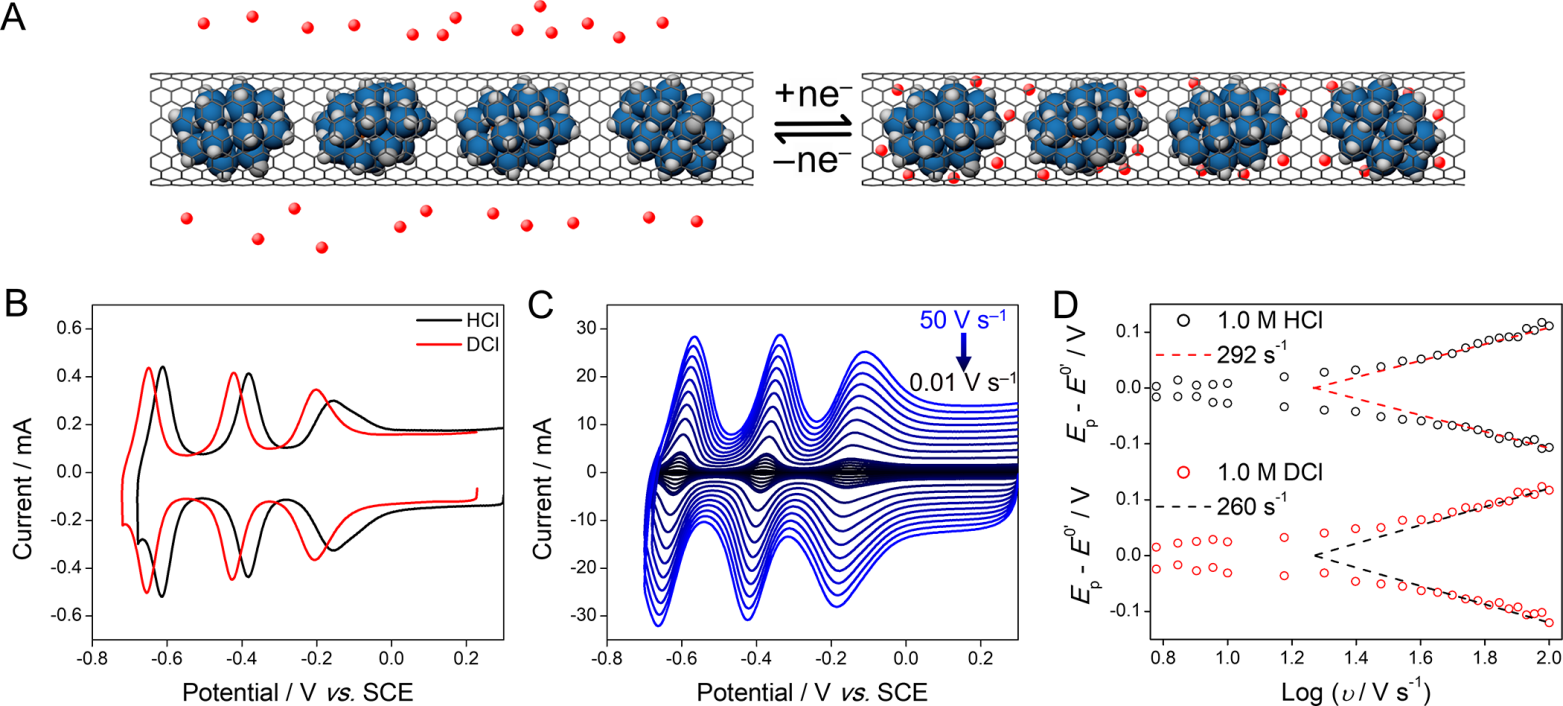
(A) Transport of protons (shown as red point charges) through the graphenic wall of the SWNT that accompanies the electron transfer to and from the encapsulated POMs. (B) Cyclic voltammograms of {P_2_W_18_}@SWNT recorded using 1.0 M HCl as supporting electrolyte (black trace) and 1.0 M DCl (red trace) at a scan rate of 100 mV s^–1^. (C) Cyclic voltammograms of {P_2_W_18_}@SWNT recorded at various scan rates (0.01–50 V s^–1^) in 1.0 M HCl. (D) plots of *E*_p_ – *E*^0^′ vs log υ for a {P_2_W_18_}@SWNT film recorded in 1.0 M HCl and 1.0 M DCl (labeled inset). All voltammograms were recorded using a glassy carbon working electrode, an SCE reference electrode, and a glassy carbon counter electrode.

To test whether access of electrolyte cations along the main axis of the SWNTs was a significant route for ion transport, the ends of the SWNT were plugged by encapsulating C_60_-fullerene within the {P_2_W_18_}@SWNT open ends, yielding ⊂C_60_{P_2_W_18_}@SWNT (Figure 3A). This was achieved by drop-casting C_60_-fullerene-saturated toluene onto a {P_2_W_18_}@SWNT-modified glassy-carbon electrode. The van der Waals diameter of C_60_ (1 nm) leaves virtually no gap for ions to pass when encapsulated within the SWNT (Figure 3A, inset).^28^Figure 3C,D shows TEM images of SWNT and {P_2_W_18_}@SWNT films subjected to the plugging process, demonstrating the effectiveness of the methodology. Figure 3E shows an image of the unplugged {P_2_W_18_}@SWNT for comparison. CVs of ⊂C_60_{P_2_W_18_}@SWNT recorded using 1.0 M HCl as an electrolyte (Figure 3B) showed little difference to those of the “unplugged” material, with minor positive shifts of 8, 9, and 9 mV in *E*_mid_ of couples I, II, and III, respectively. No significant change in the charge passed was observed, suggesting that restricting the transport of cations through the open SWNT ends and through the internal channel had little effect on charge balancing within the system. In contrast, when carrying out voltammetry in 1.0 M Li_2_SO_4_, the charge passed during redox cycling of ⊂C_60_{P_2_W_18_}@SWNT was about 50% lower than for the unplugged material (Figure S2 Supporting Information). These observations show that redox of the encapsulated POMs was supported in acidic electrolytes, even when ion access along the SWNT channel was effectively blocked. Therefore, transport of protons through the pristine SWNT sidewall likely dominated local mass transport (Figure 4A), a finding that correlates well with the pioneering work of Lozada-Hidalgo, Geim and co-workers on the transport of protons across graphene monolayers.^1,8^

To explore the nature of the proton transfer further and explore whether the mass of the cations played a significant role during charge balancing, CVs of {P_2_W_18_}@SWNT were recorded using 1.0 M HCl and DCl as electrolytes (Figure 4B). Both showed redox couples I, II, and III, but the *E*_mid_ values were shifted negatively by 44, 43, and 39 mV, respectively, in DCl relative to those in HCl, due to the lower acidity of DCl associated with the lower zero-point energy of deuterons.^44^ Δ*E*_p_ values of 6, 4, and 4 mV were observed for couples I, II, and III, respectively, in 1.0 M DCl, indicating that redox of the encapsulated POMs could still be supported when deuterons were the mobile cations.

Varying υ between 0.01 and 100 V s^–1^ (Figure 4C) can allow calculation of the standard heterogeneous electron-transfer rate constants, *k*^0^, using the Laviron equation.^45−48^ The surface-confined nature of electron transfer within the system and strong adsorption of both the reduced and oxidized species makes {P_2_W_18_}@SWNT an excellent system for study with this method. Eq 1 shows how the peak potential, *E*_p_, is related to υ for a system containing a surface-confined redox species:
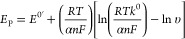
1where *E*^0^′ is the formal potential, *R* is the ideal gas constant, *T* is the absolute temperature, α is the transfer coefficient, *n* is the number of electrons transferred, and *F* is the Faraday constant. Graphs of *E*_p_ – *E*^0^′ vs log υ (so-called trumpet plots) were produced from voltammetric data for couple I in each case and showed very small *E*_p_ – *E*^0^′ values at low υ values for {P_2_W_18_}@SWNT (Figure 4D). Upon increasing υ, *E*_p_ – *E*^0^′ for the reduction and oxidation processes increased linearly above approximately log υ = 1.5. Determination of *k*^0^ from the linear gradients given by the dashed lines in Figure 4D (see Supporting Information for details) gave average *k*^0^ values of 292 ± 6 and 260 ± 1 s^–1^ for couple I when cycling in 1.0 M HCl and 1.0 M DCl, respectively. These values are lower than the values of 500 and 800 s^–1^ determined recently by Proust and co-workers, who studied monolayers of covalently-grafted POMs on electrode surfaces in organic electrolytes,^46,47^ and 2–5 orders of magnitude lower than for bridged organometallic complexes grafted onto electrode surfaces.^49,50^ The Laviron analysis of ⊂C_60_{P_2_W_18_}@SWNT yielded *k*^0^ values of 214 ± 87 and 137 ± 27 s^–1^ for couple I in 1.0 M HCl and 1.0 M DCl, respectively, demonstrating that electron transfer rates were affected when mass transport along the SWNT channel was inhibited by plugging the ends of the SWNTs but remained high even when the only route of ion transport was through the graphenic lattice of the SWNTs. Perhaps, most notably, the ratio of *k*^0^ values determined using the 1.0 M deuterated and nondeuterated acids (*k*^0^_H_/*k*^0^_D_) is 1.12 and 1.56 for unplugged and plugged {P_2_W_18_}@SWNT, respectively. These ratios are lower than reported for H^+^ and D^+^ transport across graphene, indicating that the rate of the redox switching in our system is not limited by the rate of hydron (protons or deuterons) transport across the sp^2^ framework.^10^ This observation may be due to the fact that ion transport during the electrochemical cycling of our system occurs not only across the SWNT sidewalls but also throughout the 3D {P_2_W_18_}@SWNT films.

## Conclusions

We have demonstrated the use of electrochemical methods to study the transport of protons and deuterons across the graphenic lattice of SWNTs. Cyclic voltammetry of SWNTs containing nanoencapsulated polyoxometalate anions ([P_2_W_18_O_62_]^6–^) in various aqueous electrolytes shows that sustained redox of the guest species is possible in the presence of protons or deuterons. However, in supporting electrolytes containing Li^+^ or Na^+^ as cations, the electrochemical signal of the guest species decays rapidly. Plugging the ends of the SWNTs to inhibit the transport of ions along the main SWNT channels confirms that transport of protons and deuterons through the graphenic lattice of the SWNTs occurs during electrochemical cycling of the nanoencapsulated redox species. These observations demonstrate that the ion-transport properties of 2D graphene extend to the curved graphenic walls of carbon nanotubes, with potential implications for the development of materials based on nanoconfined redox species and nanocarbon frameworks. The advantages of nanoconfinement of active molecules in confined spaces, which can include novel types of reactivity and stability, can potentially be imparted to electrochemically active materials that require facile transport of charge-balancing counterions during operation.

## Experimental Section

All reagents were purchased and used as received from Sigma-Aldrich, Merck, or Acros Organics. SWNT-P2 was used as received from Carbon Solutions Inc. K_6_[P_2_W_18_O_62_] and {P_2_W_18_}@SWNT were synthesized as reported previously.^38,51^ Cyclic voltammetry was performed using a CH Instruments 1140 potentiostat and a BioLogic SP-300 potentiostat using a three-electrode cell, comprising a glassy carbon working electrode (0.071 cm^2^) and glassy carbon counter electrode. A saturated calomel electrode (SCE) was used as the reference electrode, while an SCE-containing D_2_O as the solvent was used for voltammetry of deuterated media. Unless otherwise stated, all potentials are reported relative to that of the SCE (3 M KCl in D_2_O references adjusted as measured). {P_2_W_18_}@SWNT was sonicated for 15 min in a DMF “ink” (10 mg/mL), after which 8 μL of the resulting suspension was deposited onto the GC electrode and allowed to dry in the air. Plugging of {P_2_W_18_}@SWNT-modified electrodes was carried out by depositing 8 μL of a C_60_-fullerene-saturated toluene solution (approximately 2.2 mg/mL) onto the modified electrode, followed by soaking the electrode in fresh toluene briefly to remove unencapsulated fullerene.

### Imaging Methods

TEM images were acquired using a JEOL 2100F field emission microscope with an accelerating voltage of 100 kV. Samples were prepared by dispersing in isopropyl alcohol and drop casting onto copper grids covered with a “lacey” carbon film. All TEM images were processed using Gatan Digital Micrograph, and quoted distances were measured by drawing a line profile and measuring the electron intensity histogram.

## Supporting Information Available

Description of the determination of *k*^0^ values; plots of peak current versus scan rate (Figure S1); and cyclic voltammograms (Figure S2) (PDF)
