# Supplementary material for: Voltammetric Evidence of Proton Transport through the Sidewalls of Single-Walled Carbon Nanotubes
Source: J Am Chem Soc. 2023 Mar 28;145(16):9052–8. doi: 10.1021/jacs.3c00554 (PMC10141399; doi:10.1021/jacs.3c00554)
Supplement: Supplementary file 1 — ja3c00554_si_001.pdf [file ja3c00554_si_001.pdf]

Supporting Information

Voltammetric Evidence of Proton Transport Through the Sidewalls of Single-walled Carbon Nanotubes

Jack W. Jordan,[a,b] Beth Mortiboy,[a] Andrei N. Khlobystov,[c] Lee R. Johnson,[a,b] Graham N. Newton,*[a,b] Darren A. Walsh*[a,b]

[a] Nottingham Applied Materials and Interfaces (NAMI) Group

GSK Carbon Neutral Laboratories for Sustainable Chemistry

School of Chemistry, University of Nottingham

Nottingham, NG7 2TU, UK

*graham.newton@nottingham.ac.uk

*darren.walsh@nottingham.ac.uk

[b] The Faraday Institution

Quad One, Harwell Science and Innovation Campus

Didcot, OX11 0RA, UK

[c] School of Chemistry

University of Nottingham

Nottingham, NG7 2RD, UK

Determination of k^0

$E_p - E^{0\prime}$ is related to υ by:

$$E_p - E^{0\prime} = \left(\frac{RT}{\alpha nF}\right)\left[\ln\left(\frac{2.3RTk^0}{\alpha nF}\right) - \ln\upsilon\right]$$

Therefore, a graph of the anodic and cathodic $E_p - E^{0\prime}$ values *vs.* log υ should yield two straight lines with the following slopes:

$$gradient = \frac{-2.3RT}{\alpha nF}\ (cathodic) \qquad gradient = \frac{2.3RT}{(1-\alpha)nF}\ (anodic)$$

Determining υ from the intercept when $E_p - E^{0\prime} = 0$, allows us to solve for k^0, according to:

$$k^0 = \frac{\alpha nF\upsilon}{RT}$$

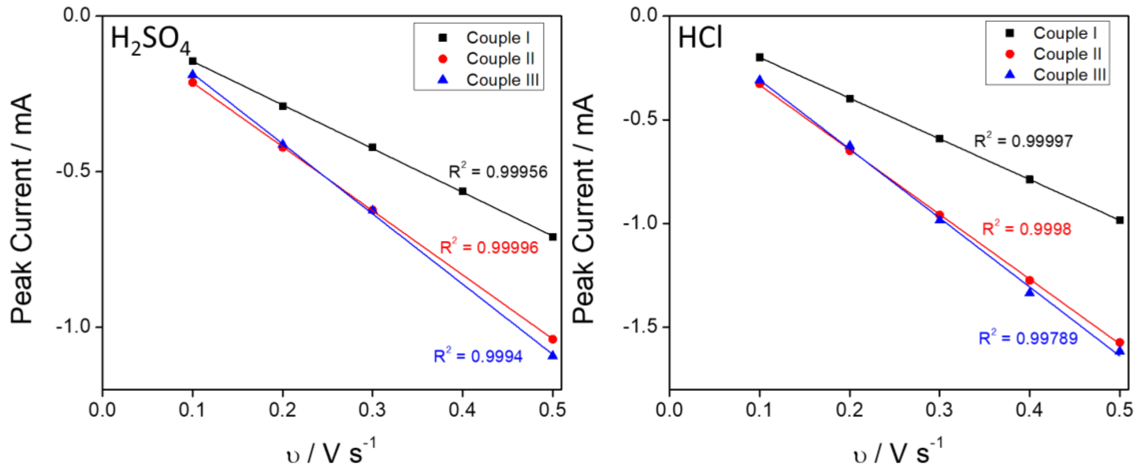

Figure S1. Plots of peak current versus scan rate for the reduction processes of redox couples I, II, and III $\{P_2W_{18}\}$@SWNT in 1.0 M H_2SO_4 and 1.0 M HCl (labelled inset). R^2 values are inset. Voltammetric data were recorded using a glassy carbon working electrode, SCE reference electrode, and glassy carbon counter electrode.

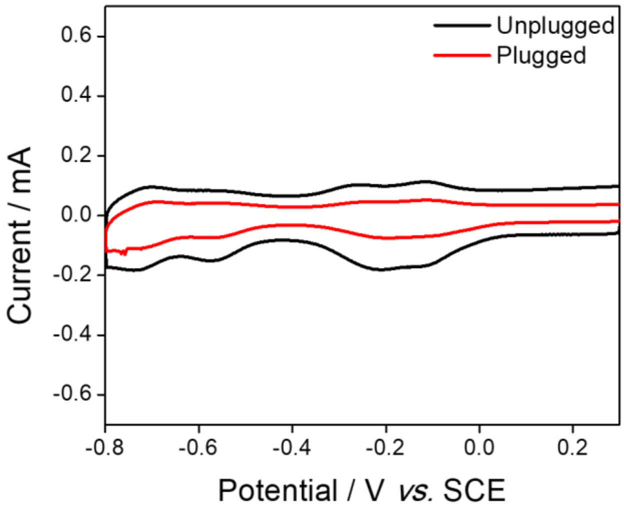

Figure S2. Cyclic voltammograms of $\{P_2W_{18}\}$@SWNT and $\subset C_{60}\{P_2W_{18}\}$@SWNT recorded using 1.0 M Li_2SO_4 as electrolyte. Voltammograms were recorded using a glassy carbon working electrode, SCE reference electrode, a glassy carbon counter electrode, and a scan rate of 100 mV s^{-1}.
